# The quest for an optimal alpha

**DOI:** 10.1371/journal.pone.0208631

**Published:** 2019-01-02

**Authors:** Jeff Miller, Rolf Ulrich

**Affiliations:** 1 Department of Psychology, University of Otago, Dunedin, New Zealand; 2 Research Group for Cognition and Perception, Department of Psychology, University of Tübingen, Tübingen, Germany; University of North Carolina at Chapel Hill, UNITED STATES

## Abstract

Researchers who analyze data within the framework of null hypothesis significance testing must choose a critical “alpha” level, *α*, to use as a cutoff for deciding whether a given set of data demonstrates the presence of a particular effect. In most fields, *α* = 0.05 has traditionally been used as the standard cutoff. Many researchers have recently argued for a change to a more stringent evidence cutoff such as *α* = 0.01, 0.005, or 0.001, noting that this change would tend to reduce the rate of false positives, which are of growing concern in many research areas. Other researchers oppose this proposed change, however, because it would correspondingly tend to increase the rate of false negatives. We show how a simple statistical model can be used to explore the quantitative tradeoff between reducing false positives and increasing false negatives. In particular, the model shows how the optimal *α* level depends on numerous characteristics of the research area, and it reveals that although *α* = 0.05 would indeed be approximately the optimal value in some realistic situations, the optimal *α* could actually be substantially larger or smaller in other situations. The importance of the model lies in making it clear what characteristics of the research area have to be specified to make a principled argument for using one *α* level rather than another, and the model thereby provides a blueprint for researchers seeking to justify a particular *α* level.

## Introduction

The statistical methods traditionally used in psychology, medicine, economics, and many other empirical disciplines have recently come under intense scrutiny, primarily because a large number of published results appear to reflect chance findings—so-called *false positives* (FPs)—rather than replicable scientific phenomena [1–5]. There have long been concerns that FP rates might be unacceptably high due to a combination of publication bias [6], the rareness of true effects within certain research areas [7, 8], and inappropriate data analysis methods [2, 4, 9], as well as outright fraud [10]. Such concerns have recently been intensified by empirical evidence, both from surveys indicating that researchers do engage in practices known to increase FP rates (e.g., [11–13]; but see [14]), and from the detection of statistical signs that published results have been contaminated by such practices [15–18]. Most tellingly, various systematic attempts to replicate published results have ended with disappointingly low replication rates (e.g., [19–22]; but see [23]). In light of this evidence, numerous strategies for reducing the worryingly high rate of published FPs have been proposed [24–26], and there is good agreement that common scientific practices and processes can be improved in a number of ways.

One particular strategy for reducing the rate of FPs is at present hotly debated; namely, the strategy of reducing the critical *α* level for concluding that an effect is real. In contrast to the *α* = 0.05 level that was suggested by Fisher [27] and has been standard for many years [28], various authors have recently argued that much smaller *α* levels should be used [29–32]. For example, in an article with 72 authors, Benjamin et al. argued that researchers should change to using *α* = 0.005 rather than *α* = 0.05, because this change in *α* would be expected to reduce the rate of FPs [33]. Benjamin et al. also argued that “a change to P = 0.005…would immediately improve the reproducibility of scientific research in many fields” (p. 6). Contrary to this claim, however, changing from *α* = 0.05 to *α* = 0.005 can actually decrease the probability of a successful replication if the same *α* level is used for all studies. As a numerical example, consider the case of a two-sample *t*-test with *n* = 60 participants per sample and a true effect size of *d* = 0.5 that is present with a base rate of *π* = 0.3. The probability of successful replication would be 0.76 for *α* = 0.05 but only 0.54 for *α* = 0.005, illustrating that decreasing *α* can decrease the probability of successful replication.

Others, however, have argued against the move to reduce *α*. In a reply to Benjamin et al. signed by 88 authors, Lakens et al. noted that a reduction in *α* would also have various negative consequences [34]. Perhaps most importantly, decreasing *α* would decrease statistical power and thereby increase the rate of *false negatives* (FNs)—that is, the proportion of studies that fail to find conclusive evidence for an effect that actually is present [14, 35].

Statistical significance at the conventionally agreed *α* level is a major factor in determining what findings are regarded as having been firmly-enough established to warrant publication [36–38], so it is clearly very important to determine the optimal level. The current debate about *α*, however, illustrates the complexity of determining its optimal value [39, 40]. Indeed, there are good reasons to believe that no single *α* level is optimal for all research contexts [34], and in some contexts there are strong arguments for increasing the *α* level to a value larger than 0.05 [41]. At this point, the only agreement concerning the choice of *α* level is that researchers within a given area should make it carefully—but how are they to do that?

The purpose of the present article is to show exactly what is necessary to provide a principled justification for a particular *α* level. Using well-known principles of statistical decision theory [42] within the context of a simple mathematical model suggested previously [43], we identify the parameters of a research scenario that must be considered when choosing the optimal *α* level for that scenario, and we indicate how the effects of those parameters can be combined quantitatively. To illustrate this model, we then show how it can be used to determine whether *α* = 0.005 or *α* = 0.05 would work better within a particular research scenario, given the required information about that scenario’s parameters. We conclude that no definitive case for any particular *α* level has yet been made, because advocates of particular *α* levels have never specified—even approximately—the key research parameters whose values are needed to identify the optimal *α*. In addition, although it is universally acknowledged that many factors must be taken into account when choosing *α*, no quantitative models have been used to compare the overall costs and benefits of different *α* levels, with proponents of different viewpoints relying instead on rather subjective justifications such as “We believe that efficiency gains [of a change to *α* = 0.005] would far outweigh losses” (p. 8, [33]). To provide an objective basis for the debate, in the following sections we show how a simple model based on the principles of statistical decision theory can be used to quantify the costs and benefits of various *α* levels, as is required for researchers to choose the optimal one.

## 1 Statistical fundamentals

The tradeoff between FPs and FNs can be formalized within a simple model in which the overall research scenario is regarded as a collection of studies testing different null hypotheses [21]. Some null hypotheses are false, and we refer to the proportion of these as the *base rate* of true effects, denoted *π*. The remaining null hypotheses, with proportion 1 − *π*, are true, at least to a good approximation. In each study, the null hypothesis is rejected or not rejected, depending on whether a statistical analysis produces significant results at the chosen *α* level. Thus, studies testing true null hypotheses may produce either FPs or true negative (TN) outcomes, whereas studies testing false null hypotheses may produce either FNs or true positive (TP) outcomes. The probabilities of these four outcomes are

$$\begin{array}{ccc} \mathrm{Pr}(FP) & = & (1-\pi )\cdot \alpha \end{array}$$ (1)

$$\begin{array}{ccc} \mathrm{Pr}(TN) & = & \left(1-\pi )\cdot (1-\alpha \right) \end{array}$$ (2)

$$\begin{array}{ccc} \mathrm{Pr}(FN) & = & \pi \cdot \beta \end{array}$$ (3)

$$\begin{array}{ccc} \mathrm{Pr}(TP) & = & \pi \cdot (1-\beta ), \end{array}$$ (4)

where 1 − *β* is the statistical power of the test of each false null hypothesis. This power depends on the *α* level, the size of the true effect, *d*, and on the sample size, *n*_*s*_. The rate of false positives (*R*_*fp*_) and rate of false negatives (*R*_*fn*_) are then

$$\begin{array}{ccc} R_{fp} & = & \frac{\mathrm{Pr}(FP)}{\mathrm{Pr}(FP)+\mathrm{Pr}(TP)} \end{array}$$ (5)

$$\begin{array}{ccc} R_{fn} & = & \frac{\mathrm{Pr}(FN)}{\mathrm{Pr}(FN)+\mathrm{Pr}(TN)}. \end{array}$$ (6)

**Fig 1:**
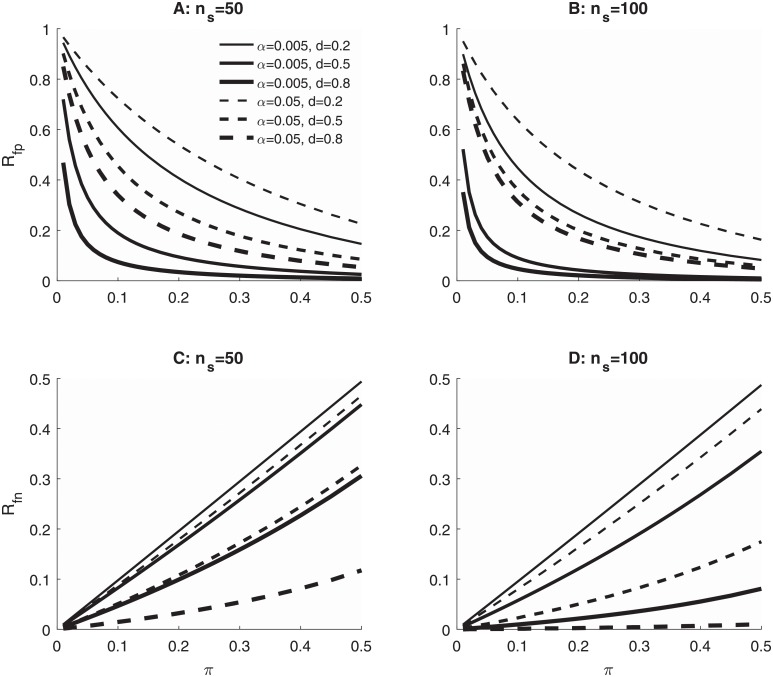
Rates of false positives and false negatives in different research scenarios. *R*_*fp*_ (A, B) and *R*_*fn*_ (C, D) as functions of the *α* level used in testing the null hypothesis, the base rate of true effects across studies (*π*), the size of the true effects when they are present (*d*), and the total sample size of the study (*n*_*s*_). Computations were carried out for studies analyzed with one-tailed two-sample *t*-tests (i.e., samples of *n*_*s*_/2 in each group). Effect size *d* is the difference between the group means divided by the common within-group standard deviation, *d* = (*μ*_2_ − *μ*_1_)/*σ*.

Fig 1 illustrates how these rates change with the researcher’s *α* level, showing results for two different sample sizes (*n*_*s*_), three different effect sizes (*d*), and a wide range of base rates (*π*). Critically, for every combination of sample size, effect size, and base rate, the rate of FPs is higher with *α* = 0.05 than with *α* = 0.005. In contrast, the rate of FNs is always higher for *α* = 0.005 than for *α* = 0.05. Thus, these two types of decision errors trade off against one another as *α* changes, and a quantitative model incorporating the frequencies and costs of these errors must be used to choose *α*.

## 2 Choosing between *α* = 0.05 and *α* = 0.005

The costs and benefits of using alternative *α* levels can be assessed quantitatively using standard decision-theory methods [42, 43]. Any given empirical study will produce one of four possible outcomes (i.e., TP, FP, TN, FN) with the probabilities just described [i.e., Pr(*TP*), Pr(*FP*), Pr(*TN*), Pr(*FN*)]. Each of the four outcomes has its own individual *informational payoff value*, and these values may be denoted as $P_{tp}$, $P_{fp}$, $P_{tn}$, and $P_{fn}$, respectively. The units of these informational payoffs are entirely arbitrary, so it is convenient to fix $P_{tp}=1$ and scale the other payoffs relative to that. On this scale, for example, $P_{fp}=-2$ means that the informational harm to a research area of one FP exactly offsets the informational benefit of two TPs. These individual outcome payoffs would vary across research areas, and it would usually only be possible to estimate them rather subjectively. For example, within a certain research area, researchers might regard the information gains associated with TPs and TNs as representing payoffs of +1 and +0.2, whereas the losses associated with FPs and FNs could be estimated to be -2 and -0.5. The average informational payoff for a single study is simply the weighted average of the four individual outcome payoffs:

$$\begin{array}{c} P_{1}=\mathrm{Pr}\left(TP\right)\cdot P_{tp}+\mathrm{Pr}\left(FP\right)\cdot P_{fp}+\mathrm{Pr}\left(TN\right)\cdot P_{tn}+\mathrm{Pr}\left(FN\right)\cdot P_{fn}. \end{array}$$ (7)

Finally, the total payoff associated with all of the studies conducted within the research scenario is

$$\begin{array}{c} P_{T}=k\cdot P_{1}, \end{array}$$ (8)

where *k* is the number of studies conducted within that scenario.

Researchers in a given research scenario must make two separate choices that could influence their expected payoffs, and their goal is to make these choices in a manner that will maximize that payoff. One choice is the sample size (*n*_*s*_) to be used in each study. Larger studies have greater statistical power (1 − *β*), but they are more time-consuming and expensive to conduct, so increasing *n*_*s*_ decreases the total number of studies (*k*) that can be conducted. The other choice is the *α* level, which is currently being debated. For simplicity, we and others discuss the choice of *α* level as if it were entirely up to the researchers. In practice, though, the researchers’ choice of *α* level may be heavily constrained by the editorial policies of the journals in which they hope to publish their results [36]. In principle, though, the researchers’ problem is to choose the particular values of *n*_*s*_ and *α* that produce the maximum total payoff, $P_{T}$.

**Fig 2:**
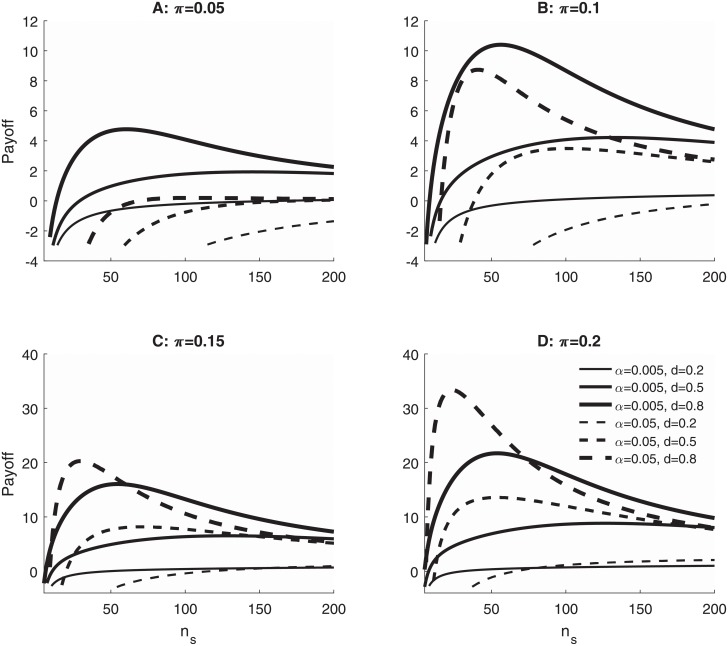
Expected payoffs in different research scenarios. Expected total payoff, ($E[P_{T}]$, ordinate) as a function of *α* level and sample size (*n*_*s*_) for research scenarios differing in the size of a true effect when it is present (*d* = 0.2, 0.5, or 0.8) and in the base rate probability that the true effect is present (*π*). The range of base rates, 0.05–0.20, spans approximately the range 0.024–0.167 used by Benjamin et al. in their computational examples [33]. Payoffs were computed from Eq 8 using individual outcome payoffs of $P_{tp}=1$, $P_{fp}=-1$, $P_{tn}=0$, and $P_{fn}=0$ and assuming a total sample size of 10, 000 across all studies (i.e., *k* = 10, 000/*n*_*s*_). Computations were carried out for studies analyzed with one-tailed two-sample *t*-tests (i.e., samples of *n*_*s*_/2 in each group). Effect size *d* is the difference between the group means divided by the common within-group standard deviation, *d* = (*μ*_2_ − *μ*_1_)/*σ*.

Fig 2 illustrates the consequences of the researchers’ choices by showing the expected total payoff as a function of sample size (*n*_*s*_) and *α* level for several example research scenarios differing in the base rate (*π*) and size (*d*) of true effects. Researchers seek to maximize their payoff, of course, so they would be advised to use *α* = 0.005 with any combination of parameters (i.e., *π*, *d*, and *n*_*s*_) for which the solid line is above the dashed line, but to use *α* = 0.05 with combinations for which the solid line is below the dashed line. In addition, though, researchers can choose the sample size, so they should also choose the sample size that leads to the highest payoff. In Fig 2B with *π* = 0.1 and *d* = 0.5, for example, the highest possible expected payoff across all sample sizes is approximately 4.22, which is obtained with *α* = 0.005 and *n*_*s*_ = 135. Thus, *α* = 0.005 is preferable to *α* = 0.05 in this situation. In contrast, in Fig 2C with *π* = 0.15 and *d* = 0.5, the highest payoff is approximately 8.18, obtained with *α* = 0.05 and *n*_*s*_ = 70, so *α* = 0.05 would be preferable in this case.

The contrast between the two numerical examples just discussed has profound implications for the current controversy over the best choice of *α* level. If *α* = 0.005 is better when the base rate is less than *π* = 0.10 but *α* = 0.05 is better when the base rate is greater than *π* = 0.15, then it follows that *researchers must know the base rate of true effects*—at least approximately—before they can choose the right *α* level. This shows that advocates of a particular *α* level should specify the range of base rates being assumed and acknowledge that other *α* levels would be appropriate for other base rates. To our knowledge this has never been done, although Benjamin et al. did support their argument for *α* = 0.005 partly by presenting evidence for a base rate of approximately 10% [33].

For the present purposes, another important point illustrated by Fig 2 is that the choice between *α* = 0.05 and *α* = 0.005 can depend on the sample size. For example, with *d* = 0.8 in Fig 2C and 2D, the payoff is higher for *α* = 0.05 at some sample sizes but higher for *α* = 0.005 at other sample sizes (i.e., the thick solid and dashed lines cross within both panels). This dependence of *α* preference on sample size is also quite relevant to the debate about *α* levels. It implies that there is no single best *α* across all sample sizes—even within a given research scenario. Again, this implies that advocates of a particular *α* level must specify the sample sizes to which their recommendations apply as well as the base rates of true effects.

**Fig 3:**
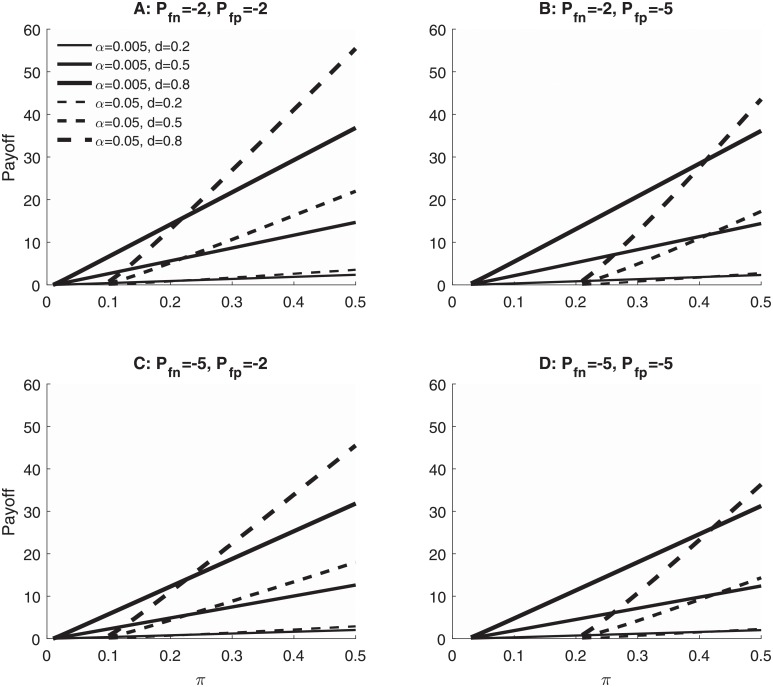
Maximum expected payoffs at optimal sample sizes. The maximum expected total payoff (ordinate), taken across all possible values of *n*_*s*_, that could be achieved for each combination of *α* level, base rate probability that a true effect is present (*π*), the size of the effect when it is present (*d* = 0.2, 0.5, or 0.8), the payoff associated with false positives ($P_{fp}$), and the payoff associated with false negatives ($P_{fn}$). Payoffs were computed as in Fig 2 using individual outcome payoffs of $P_{tp}=1$ and $P_{tn}=0$. Computations used the same statistical test and definition of *d* as in Fig 2.

Finally, the choice between *α* = 0.05 and *α* = 0.005 also depends strongly on the exact quantitative payoffs associated with TPs, FPs, TNs, and FNs. To illustrate that, Fig 3 shows how the total payoffs available with *α* = 0.05 and *α* = 0.005 depend on the individual payoffs associated with false positives ($P_{fp}$) and false negatives ($P_{fn}$), as well as the base rate of true effects (*π*), and the size of the effect when it is present (*d*). Each plotted total payoff value is the maximum (i.e., across all possible values of sample size, *n*_*s*_) for the indicated combination of parameters, so the figure is computed assuming that researchers have chosen the optimal sample size for each scenario. Again, *α* = 0.005 should be preferred with any combination of parameters for which the solid line is above the dashed line, but it is better to use *α* = 0.05 with combinations for which the solid line is below the dashed line. Comparing the different panels, it is easy to see that the cross-over points for *α* = 0.05 versus *α* = 0.005 depend heavily on the individual payoffs associated with the various outcomes. As was true with base rates and sample sizes, this implies that advocates of a particular *α* level must provide the values of individual outcomes to which their recommendations apply and acknowledge that other *α* levels could be appropriate for other values.

Several general lessons about the relative merits of *α* = 0.05 and *α* = 0.005 can be seen in Fig 3, and these help to sharpen intuitions about exactly when each of the two *α* levels—together with its optimal sample size—would be preferable. First, for the values of $P_{fp}$ and $P_{fn}$ examined here, *α* = 0.005 yields larger payoffs when the base rate of true effects is smaller than approximately 0.1, whereas *α* = 0.05 yields larger payoffs when the base rate is larger than approximately 0.4, which provides some boundaries for the use of each *α* level. Second, for intermediate base rates (i.e., 0.1 < *π* < 0.4), the cross-over points at which researchers should switch between the two *α* levels are quite sensitive to the cost associated with FPs, as can be seen by comparing Fig 3A with 3B. Qualitatively, this is exactly as expected: To the extent that FPs are relatively costly (e.g., $P_{fp}=-5$ as opposed to $P_{fp}=-2$), *α* = 0.005 tends to be preferred over *α* = 0.05 because it produces fewer of them. Third, and perhaps somewhat surprisingly, the 0.05/0.005 cross-over points are not very sensitive to the cost associated with FNs, as can be seen by comparing Fig 3A with 3C or Fig 3B with 3D. This is presumably because the base rate of true effects, *π*, is low, which means that FNs are rare so their cost is not too important. The situation would be reversed if the base rate were high, because in that case FPs would be rare and their cost could be relatively unimportant compared to that of FNs. Fourth, and also somewhat surprisingly, the 0.05/0.005 cross-over points do not seem to be affected much by the true effect size *d*. In Fig 3A, for example, the solid and dashed lines cross at a base rate of about *π* = 0.23 for all three *d* values, and the cross-over base rates are also fairly constant across *d* values in Fig 3B–3D. Illustrative computations in S1 Appendix “Supplementary analysis of other possible *α* levels” show even more clearly that the optimal *α* level depends very little on the true effect size *d*. In summary, then, the optimal *α* value depends most heavily on the base rate of true effects and secondarily on the relative payoffs of the individual outcomes, especially on the cost of an FP when the base rate is low and on the cost of an FN when the base rate is high.

## 3 General discussion

By viewing empirical hypothesis testing in a decision-theoretic framework with fixed total resources (i.e., fixed total participants tested, *k* ⋅ *n*_*s*_), it is possible to calculate precisely how researchers’ expected total scientific payoffs depend on their choices of *α* levels and sample sizes within any given research scenario. It is important to examine these total payoffs to understand which research scenario parameters must be considered and to see how the size of the payoff is jointly determined by the various parameter values. There is wide agreement that scientists in any field should consider their *α* levels carefully [33, 34, 44, 45], and it seems essential to use an objective formalism to compare the expected scientific payoffs of different *α* levels.

The present approach differs from previous statistical decision models, because these have usually considered the problem of choosing either *α* level or sample size while keeping the other value fixed. For example, the optimum choice of *α* has been investigated for a fixed sample size in terms of minimizing the expected number and/or cost of errors [39, 46]. Similarly, within the context of hypothesis testing, the sample size is usually chosen to achieve sufficient statistical power for a fixed *α* level [47]. The choice of sample size has also been analyzed outside of the hypothesis testing context, with an emphasis on maximizing overall economic, medical, or environmental benefits [48–51], but these analyses have had no clear implications for the choice of *α*.

The present approach highlights the fact that the optimal choices of *α* level and sample size depend in a complicated fashion on numerous parameters. Because *α* and sample size are the only parameters that are usually under the researchers’ control, researchers should strive to make optimal choices for them to improve the use of scientific resources [1, 52, 53]. The other parameters (i.e., *π*, *d*, $P_{tp}$, $P_{fp}$, $P_{tn}$, and $P_{fn}$) are essentially inherent in the research area and are thus outside of the researchers’ control, but their values must be considered nonetheless. The computations shown in Figs 2 and 3 reflect research scenarios in which there was either an effect of a given fixed size (i.e., *d* = 0.2, 0.5, or 0.8) or there was no effect at all. Analogous computations were carried out for scenarios in which the true effect size varied randomly, and the results were fairly similar. These computations and the differences from the present results are reported in S2 Appendix “Supplementary analysis of varying effect sizes”.

Critically, the optimal choices of *α* level and sample size depend strongly on the values of these other, “out of control” parameters. This presents a challenge for researchers who would like to determine the optimal *α* level and sample size using Eq 8, because it is essential to obtain good estimates of their values. The standard expected value model underlying Eq 8 is valuable partly because it clarifies exactly which parameters must be estimated to argue for a particular *α* level or sample size. In addition, considerable insight can be gained from total payoff computations by making rough estimates and performing “what if” calculations, as is done in many scientific areas where parameter estimates are difficult to obtain. For example, economists use models to project future economic growth and activity, despite the fact that future economic conditions (i.e., parameter values) are unknown because conditions can change abruptly. Similarly, models of global climate change and of endangered species population sizes are used to make ball-park calculations and to inform decision-makers despite major uncertainties about key parameter values.

From the present results, it appears that the base rate of true effects, *π*, is the parameter with the strongest influence on the optimal choices of *α* level and sample size (e.g., Fig 3; also see Figs A–C in S1 Appendix). In the case of *α* level, for example, switching from the current standard *α* = 0.05 to the proposed new *α* = 0.005 could very well increase payoffs in scenarios where the base rate is lower than approximately 0.1, but it could equally well decrease payoffs if the base rate is higher than approximately 0.4. For scenarios with base rates within the range of 0.1-0.4, the choice between *α* = 0.05 and *α* = 0.005 would be heavily influenced by the relative costs of FPs and FNs.

We have focussed on comparing the payoffs for *α* = 0.05 and *α* = 0.005 as two specific values currently being advocated, but these are only two of the infinitely many possible *α* levels that could be used. In principle, it is possible to determine exactly which *α* level leads to the highest expected payoff, whether it is one of these two *α* levels or not. S1 Appendix “Supplementary analysis of other possible *α* levels” illustrates how this can be done and presents illustrative computations in which the optimal *α* level varies gradually from approximately 0.001 to 0.12. Among other things, the analysis in this supplement strongly reinforces the earlier suggestions—based on Fig 3—that the base rate has a large effect on the optimal *α* level whereas the true effect size *d* has little or no effect.

It is crucial to consider the realistic base rate carefully for each research area. Mudge et al. argued on logical grounds that researchers should assume a base rate of *π* = 0.5 in the absence of any relevant information [39], but many have argued that available information hints at base rates much lower than this. In particular, when there have been attempts to replicate previous findings in an area, the base rate can be estimated from the probability of successful replication. From replication rates reported recently [22], for example, it appears that the overall base rate of true effects is approximately 10% within a broad area of experimental psychology represented by the sample of replicated studies [43, 44]. Lower replicability in certain domains of biomedical research suggest that their base rates might be even lower [2, 24]. On the other hand, base rates might be much higher in research areas where there is better prior information about the mechanisms under investigation.

Several authors have suggested that researchers can get reasonable estimates of base rate from prior area-specific knowledge [2, 8]. These estimates obviously depend a great deal on the strength of the theoretical and empirical results suggesting that the tested effect would be present (i.e., the information that led the researchers to test for the effect in the first place). When researchers only have weak grounds for suspecting the presence of a certain effect, they could use an appropriately low estimate of the base rate—perhaps 0.1 or less. In contrast, when an effect is predicted by a detailed theory that has fared well in many previous tests, it would seem appropriate to use a much larger estimate—perhaps 0.9. A high estimate would also be appropriate when, for example, the effect was a minor extension or variation of a phenomenon that had previously been clearly demonstrated. Indeed, the dependence of the base rate estimate on prior knowledge has been tacitly acknowledged by advocates of stringent *α* levels like Benjamin et al., who proposed using *α* = 0.005 only for the “discovery of new findings … [but not] for confirmatory or contradictory replications of existing claims” (p. 6, [33]).

Given the importance of estimating the base rate and given the uncertainties about how that can be done, we propose that experienced researchers can estimate the base rate in their own research areas by looking at the long-run relative frequency of getting significant results across many of their own experiments. The probability of a significant result in a study, *p*_sig_, is a function of the base rate of true effects *π* within the area, the *α* level, and statistical power 1 − *β*:

$$\begin{array}{c} p_{\text{sig}}=\pi \cdot (1-\beta )+(1-\pi )\cdot \alpha . \end{array}$$ (9)

This equation can be solved for the base rate, yielding

$$\begin{array}{c} \pi =\frac{p_{\text{sig}}-\alpha }{1-\beta -\alpha }, \end{array}$$ (10)

which allows individual researchers to estimate their true base rates from their own estimated values of *α*, power 1 − *β*, and *p*_sig_. As an example, suppose a researcher uses *α* = 0.05, conducts studies with power approximately 1 − *β* = 0.55, and finds significant results in approximately half of all studies (i.e., *p*_sig_ = 0.5). Using Eq 10, the researcher can estimate that the base rate of true effects across his or her past studies has been approximately 90%. Eq 10 can also be used to estimate a lower bound for the base rate when power is unknown. The left size of Eq 10 is minimal when *β* = 0, which implies

$$\begin{array}{c} \pi \geq \frac{p_{\text{sig}}-\alpha }{1-\alpha }. \end{array}$$ (11)

Thus, for the same researcher with *α* = 0.05 and *p*_sig_ = 0.5, the base rate must be at least 47%, regardless of the power level.

In addition to base rates, the optimal *α* levels for different scenarios also depend on the individual payoffs associated with the four possible hypothesis testing outcomes, $P_{tp}$, $P_{fp}$, $P_{tn}$, and $P_{fn}$. If the researchers working in a given field share a common sense of the approximate relative benefits and costs of these outcomes, then the agreed values would be helpful in working out the optimal *α* level. From informal discussions with colleagues, however, we believe that there are sometimes great disagreements about these values, with estimates of the FP cost varying by as much as two orders of magnitude (e.g., -2 to -200). When individual outcome payoffs are perceived so differently, it is only natural that researchers would prefer different *α* levels. Thus, the present analysis shows that a convincing case for a given *α* level must include quantitative assessments—together with supporting evidence—of the costs and benefits of the specific individual outcomes (i.e., TPs, FPs, TNs, FNs). Arguably, these assessments should come from observers who are not directly at the research coal face (e.g., journal editors, granting agencies), since the researchers themselves may have vested interests in reaching positive versus negative decisions.

In the end, the question of which *α* level researchers should use simply cannot be answered without a detailed quantitative model incorporating not only the researcher’s choices of *α* level and sample size, but also the underlying characteristics of the research scenario and the costs and benefits of reaching the different possible correct and incorrect conclusions. To that end, traditional statistical decision models can be adapted to models of the research process [43], and we suggest that advocates of any particular *α* level should use such models—in conjunction with estimates of base rates and payoffs—to give their arguments a firm objective foundation.

## Supporting information

S1 Appendix Supplementary analysis of other possible *α* levels. (PDF)

S2 Appendix Supplementary analysis of varying effect sizes. (PDF)
