# Supplementary material for: The quest for an optimal alpha
Source: PLoS One. 2019 Jan 2;14(1):e0208631. doi: 10.1371/journal.pone.0208631 (PMC6314595; doi:10.1371/journal.pone.0208631)

S1 Appendix. Supplementary analysis of other possible α levels

In principle, $\alpha = 0.05$ and $\alpha = 0.005$ are not the only two possibilities, because any number between zero and one could be chosen as the α level. Thus, in this supplement we treat α level as a continuously varying value and investigate how its optimal value depends on research scenario parameters.

The overall payoff $\mathcal{P}_T$ (Eq 8) is a function of two parameters controlled by the researcher, α and sample size, plus six parameters inherent in the research scenario, d, π, $\mathcal{P}_{tp}$, $\mathcal{P}_{fp}$, $\mathcal{P}_{tn}$, and $\mathcal{P}_{fn}$. For any given values of the research scenario parameters, standard numerical search procedures [1] can be used to find the optimal combination of α and sample size—that is, the combination leading to the highest payoff within that scenario. These search procedures treat α as a continuously varying numerical quantity and can find whatever value is optimal, even if it is something other than $\alpha = 0.05$ or $\alpha = 0.005$.

Fig A shows the optimal α levels for a variety of scenarios, and Fig B shows the corresponding optimal sample sizes. Inspection of Fig A reveals that the optimal α level depends very strongly on the base rate, π, and on the payoff associated with false positives, $\mathcal{P}_{fp}$. At one extreme, with a very low base rate (i.e., $\pi = 0.01$) and a relatively high FP cost (i.e., $\mathcal{P}_{fp} = -5$), the optimal α level is less than 0.001. In contrast, with a relatively large base rate (i.e., $\pi = 0.5$) and a relatively low FP cost (i.e., $\mathcal{P}_{fp} = -2$), the optimal α level is greater than 0.1. Given that the optimal α varies across at least two orders of magnitude, the discussion over whether to use $\alpha = 0.05$ or $\alpha = 0.005$ actually seems somewhat narrow. Perhaps more importantly, the figure shows quite clearly that researchers cannot hope to identify the appropriate α level without having good quantitative information about the base rate of true effects and about the cost of FPs. Although base rate estimates have been considered in some recent discussions of α levels [2], FP costs have not been addressed quantitatively.

Surprisingly, Fig A also shows that the optimal α level is essentially independent of the effect size, d, and it is also relatively independent of the FN cost, at least within the ranges considered here. The reasons for such independence are not entirely clear, but in principle the independence makes it more feasible to identify optimal α levels, because it reduces the amount of prior knowledge that researchers must obtain to identify these levels (i.e., d and $\mathcal{P}_{fn}$ need not be identified). In practice, however, information about d is clearly needed to identify the optimal (α, n_s) pair because it is required for choosing n_s, as will be seen next.

Fig B shows the optimal sample sizes associated with each of the optimal α levels shown in Fig A. Quite reasonably, much larger samples are needed with smaller true effect sizes, and larger samples are also needed when the base rate of true effects is low. Interestingly, at least for the conditions shown in Fig A, the sample size increases for smaller effects to an extent that produces approximately the same levels of power for all three effect sizes. Thus, not only is the optimal α level rather independent of effect size, but so is the optimal level of power. It is disconcerting, however, that the optimal sample sizes are typically 300+ for medium-sized effects; sample sizes that large are rare in many research areas where two-group comparisons are common. For repeated measures designs (i.e., one-sample t-tests), analogous computations indicate that the optimal sample sizes are approximately 25% of those shown in Fig B.

Fortunately, further investigation reveals that the choice of the *exactly* optimal α level may not be very important in practice, at least under conditions similar to those considered here (i.e., $\pi < 0.5$, $\mathcal{P}_{fp}$ and $\mathcal{P}_{fn}$ of -2 or -5). Fig C shows expected payoffs for researchers using $\alpha = 0.005$, $\alpha = 0.05$, or the exact α yielding the maximum expected payoff, each with its own optimal sample size. Critically, in these scenarios researchers can achieve nearly the same payoff by using whichever is better of $\alpha = 0.005$ of $\alpha = 0.05$, without determining the best α level even more precisely.

We also conducted a more detailed analysis to see which values of α would be optimal using a wider range of payoff values. To that end, we computed the optimal combination of α level and sample size for two-sample t-tests using all of the scenarios that could be constructed from combinations of the parameter values listed in Table A. Excluding approximately 11% degenerate scenarios in which the expected payoff was maximized by taking the minimum possible sample size (see [3], Appendix C), the optimal α levels ranged all the way from a minimum of 0.0001 to a maximum of 0.876. Interestingly, a logistic model predicted the optimal α levels from the scenario parameters fairly accurately. In this model, the to-be-predicted score was

$$Y = \log\left(\frac{\alpha_{\text{optimal}}}{1 - \alpha_{\text{optimal}}}\right), \tag{1}$$

and Y was predicted as a linear function of the parameters in Table A with a constant of -4.14 and with the slopes shown in the table, with an overall $R^2 = 0.92$.

The slopes in Table A show how the optimal values of α are affected by the values of the scenario parameters, and the sizes of the parameter effects are most clearly illustrated with numerical examples. As a baseline condition, assume $\mathcal{P}_{fp} = -2$, $\mathcal{P}_{tn} = 0$, $\mathcal{P}_{fn} = 0$, $\pi = 0.1$, and $d = 0.2$. For this condition, the model predicts an optimal $\alpha = 0.01$. If the loss associated with a false positive is reduced to $\mathcal{P}_{fp} = -1$, the predicted optimal α level increases to 0.018. The gain associated with a true negative outcome, $\mathcal{P}_{tn}$, has a much stronger influence on the predicted α_{optimal}, perhaps because there were so many opportunities for true negatives with the low base rates used in these computations. For example, a small change from the baseline condition to $\mathcal{P}_{tn} = 0.2$ increases α_{optimal} to 0.036. Similarly, α_{optimal} was quite sensitive to the base rate of true effects; changing from the baseline to $\pi = 0.3$ yields predicted $\alpha_{\text{optimal}} = 0.04$. In contrast, there seem to be relatively small influences of the effect size, d, and of the loss associated with a false negative, $\mathcal{P}_{fn}$, at least over moderate ranges of those values. For example, the predicted α_{optimal} values remain at 0.01 when there is a change from the baseline to $\mathcal{P}_{fn} = -1$ or to $d = 0.8$.

Table A. Combinations of parameter values for which optimal α levels were computed, and the slope of each parameter value in a linear model predicting the Y values shown in Equation 1.

Parameter	Values	Slope
$\mathcal{P}_{tp}$	1	
$\mathcal{P}_{fp}$	-5, -4.5, -4.0, … -0.5	0.575
$\mathcal{P}_{tn}$	0, 0.02, 0.04, … 0.10	6.463
$\mathcal{P}_{fn}$	-5, -4.5, -4.0, … 0	0.026
π	0.05, 0.1, 0.2, 0.4	6.982
d	0.2, 0.5, 0.8	0.043

References

1. Rosenbrock HH. An automatic method for finding the greatest or least value of a function. Computer Journal. 1960;3:175–184. doi:10.1093/comjnl/3.3.175.

2. Benjamin DJ, Berger JO, Johannesson M, Nosek BA, Wagenmakers EJ, Berk R, et al. Redefine statistical significance. Nature Human Behaviour. 2018;2:6–10. doi:10.1038/s41562-017-0189-z.

3. Miller JO, Ulrich R. Optimizing research payoff. Perspectives on Psychological Science. 2016;11(5):664–691. doi:10.1177/1745691616649170.

Fig A. Optimal α levels. The optimal α level, α_{optimal}, as a function of the base rate of true effects, π, the size of the true effect when it is present d, and the payoffs associated with false positives, $\mathcal{P}_{fp}$, and false negatives, $\mathcal{P}_{fn}$. Computations were carried out for studies analyzed with two-sample t-tests.

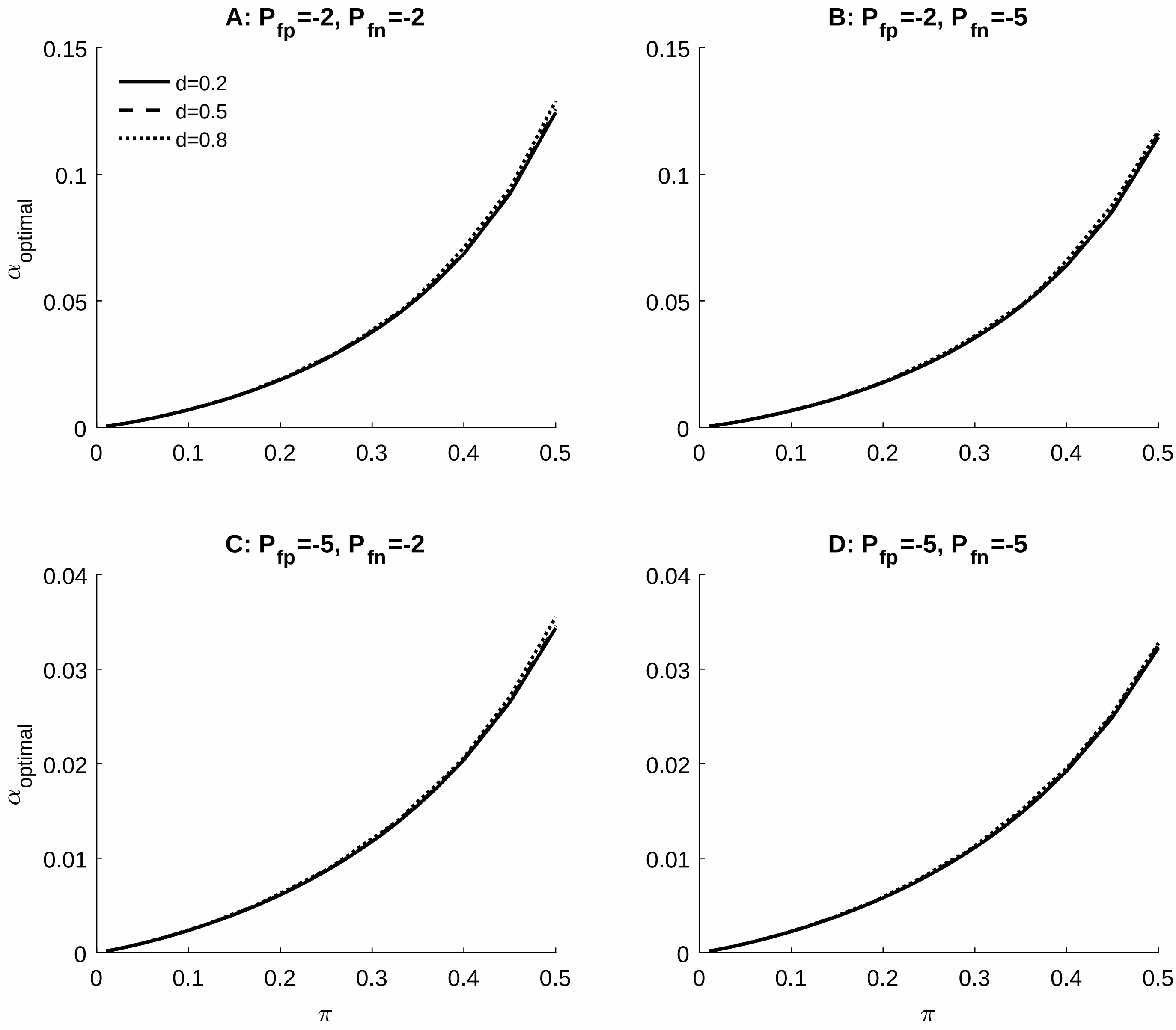

Fig B. Optimal sample sizes. Optimal sample sizes, $n_{\mathrm{s,optimal}}$, corresponding to the optimal α levels shown in Fig A.

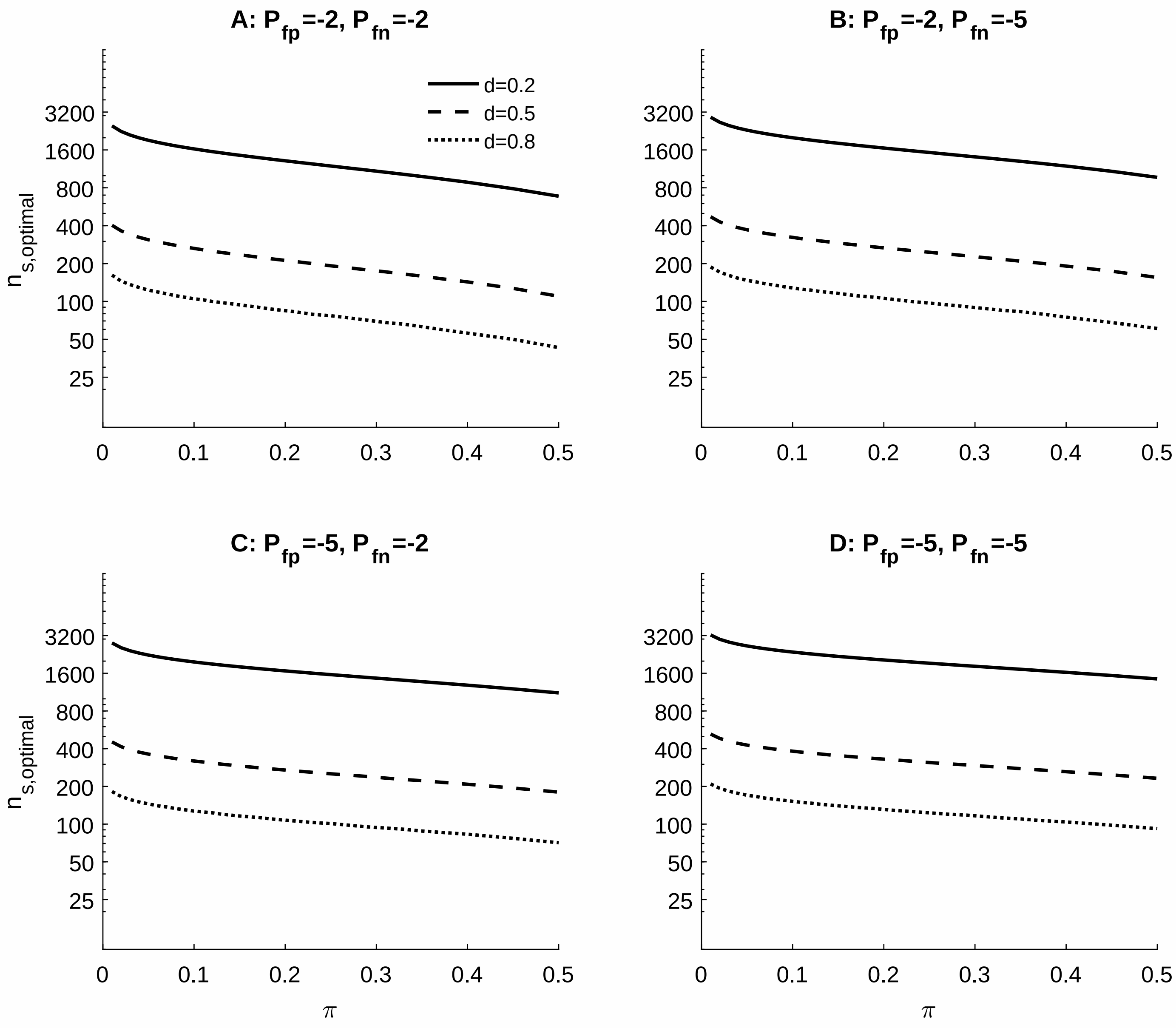

Fig C. Expected payoffs with different α levels. Expected payoff as a function of base rate, π, with $\alpha = 0.005$, $\alpha = 0.05$, or the optimal α, for scenarios differing in the payoffs associated with false positives ($\mathcal{P}_{fp}$) and false negatives ($\mathcal{P}_{fn}$). All payoffs were computed with an effect size of $d = 0.5$ and with the optimal sample size.

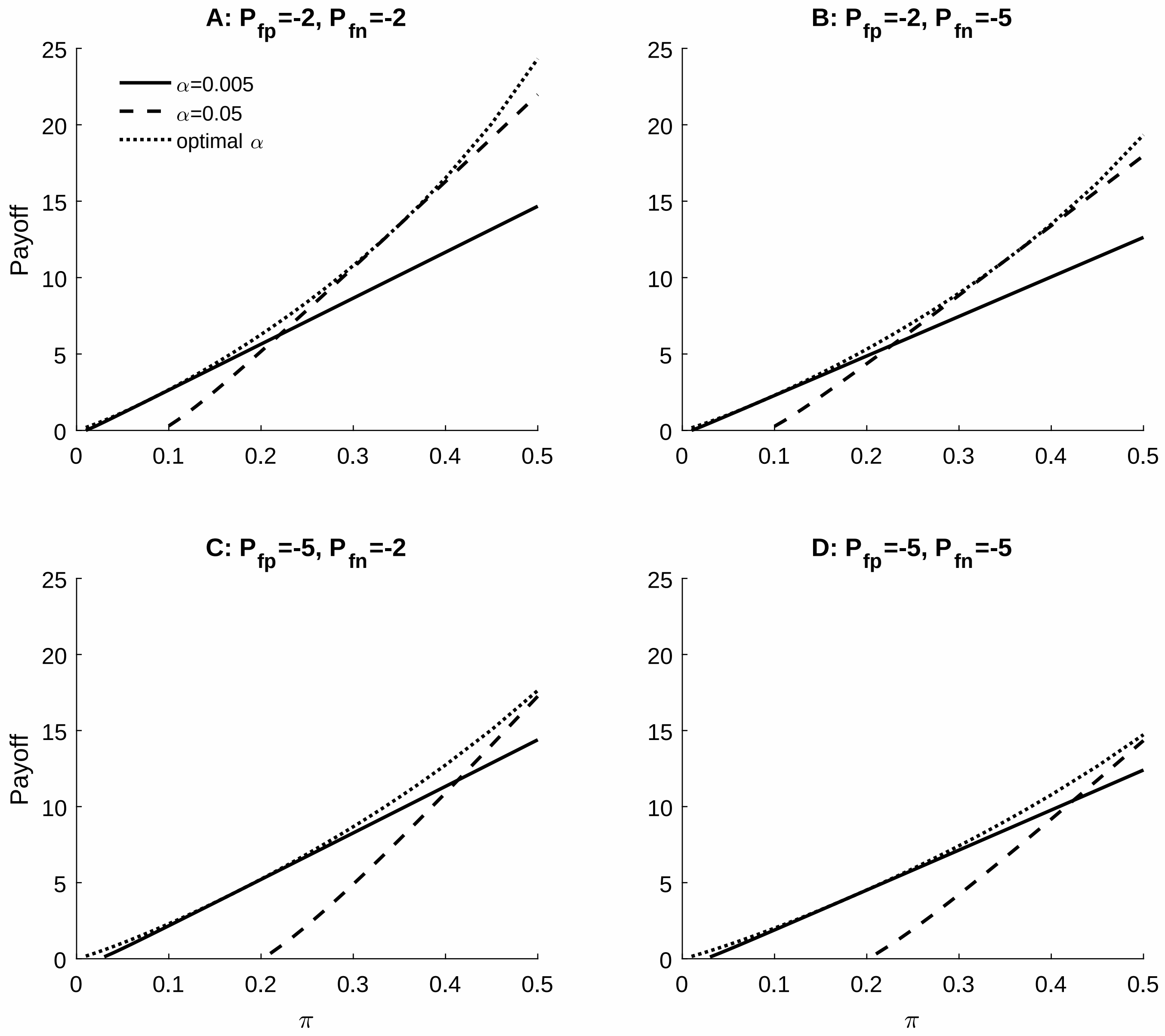

Supplement: S1 Appendix — (PDF) [file pone.0208631.s001.pdf]
