# Supplementary material for: The quest for an optimal alpha
Source: PLoS One. 2019 Jan 2;14(1):e0208631. doi: 10.1371/journal.pone.0208631 (PMC6314595; doi:10.1371/journal.pone.0208631)

S2 Appendix. Supplementary analysis of varying effect sizes

The computations in the main article were based on simplistic research scenarios in which there was either an effect of a given fixed size (i.e., $d = 0.2$, 0.5, or 0.8) or there was no effect at all. Although such scenarios are commonly analyzed because of their computational simplicity [1, 2], they are not very realistic, because true effect sizes would be expected to vary across different hypotheses that might be tested within a given scenario (e.g., different drugs might have smaller or larger true effects).

This supplement therefore reports additional computations investigating whether the patterns evident in the main article would also be present if the true effect size varied. Specifically, we examined scenarios in which the true effect sizes—if present—varied either according to a gamma distribution having a shape parameter equal to four or according to an exponential distribution. These distributions, examples of which are shown in Fig A, were chosen to represent a wide range of possible effect-size distributions. In the gamma model, there are hardly any tiny effects; instead, true effects tend to be nearer the average size, although there is still significant variation among them. In the exponential model, true effects typically tend to be quite small, but there are occasional large ones. This distribution was suggested by the results of [3], who recently estimated the empirical distribution of effect sizes using a tabulation of 26,841 t-tests published in psychological and medical journals. Collapsing across significant and non-significant results with a α cutoff of 0.05, their full empirical distribution actually appeared to have a skewed shape quite close to that of the exponential distribution. [4] reported a similarly skewed distribution of effect sizes estimated from t-tests reported in two psychological journals.

To maximize the comparability of these scenarios to the fixed-effect scenarios already considered, we adjusted the parameters of each distribution to produce a mean effect equal to one of the fixed effects already examined (i.e., $\mathrm{E}[d] = 0.2$, 0.5, or 0.8). As in the computations with fixed effect sizes, one of these—now varying—effect sizes was present with a given base rate, π, and there was no effect with probability $1 - \pi$.

In scenarios where true effect sizes vary, it seems quite plausible that the payoffs for TPs and FNs would also vary. Specifically, detecting a larger true effect would produce a greater benefit (i.e., more positive $\mathcal{P}_{tp}$), but failing detect a larger true effect would produce a greater cost (i.e., more negative $\mathcal{P}_{fn}$). To implement this idea, we let each true effect size's $\mathcal{P}_{tp}$ and $\mathcal{P}_{fn}$ be proportional to actual effect size, adjusting separate gain and loss proportionality constants for each distribution to achieve the same averages used before (i.e., $\mathrm{E}[\mathcal{P}_{tp}] = 1$ and $\mathrm{E}[\mathcal{P}_{fn}] = -2$ or -5). As before, the expected total payoff was computed for a set of such scenarios varying in base rate, $\mathcal{P}_{fp}$, and $\mathrm{E}[\mathcal{P}_{fn}]$, and these combinations were computed for different combinations of α level and sample size. These computations were carried out using the method described in Appendix B of the Supplemental Materials of [5]. Each distribution of true effect sizes was approximated using ten equally-spaced percentile points (i.e., 5%, 15%, . . . , 95%).

Fig B shows that the optimal α level differs very little across the three quite different effect-size distributions examined here. Thus, it is probably safe to ignore the precise shape of the effect size distribution as well as the mean effect size when choosing an appropriate α. In contrast, the distribution of effect sizes does influence the optimal sample size, as can be seen in Fig C. For example, when FNs are especially costly (i.e., $\mathcal{P}_{fn} = -5$), larger sample sizes are needed when effect sizes vary (i.e., Exponential or Gamma) than when they are fixed, presumably because of the need for adequate power to detect smaller-than-average effects.

References

1. Benjamin DJ, Berger JO, Johannesson M, Nosek BA, Wagenmakers EJ, Berk R, et al. Redefine statistical significance. Nature Human Behaviour. 2018;2:6–10. doi:10.1038/s41562-017-0189-z.

2. Ioannidis JPA. Contradicted and initially stronger effects in highly cited clinical research. JAMA. 2005;294(2):218–228.

3. Szucs D, Ioannidis JPA. Empirical assessment of published effect sizes and power in the recent cognitive neuroscience and psychology literature. PLoS Biology. 2017;15(3):1–18. doi:10.1371/journal.pbio.2000797.

4. Wetzels R, Matzke D, Lee MD, Rouder JN, Iverson GJ, Wagenmakers EJ. Statistical evidence in experimental psychology: An empirical comparison using 855 t tests. Perspectives on Psychological Science. 2011;6(3):291–298. doi:10.1177/1745691611406923.

5. Miller JO, Ulrich R. Optimizing research payoff. Perspectives on Psychological Science. 2016;11(5):664–691. doi:10.1177/1745691616649170.

Fig A. Example effect size distributions. Six example distributions of true effect sizes, varying in shape (i.e., exponential, gamma) and mean (0.2, 0.5, or 0.8). The effect size d is shown on the horizontal axis, and the probability density is shown on the vertical axis.

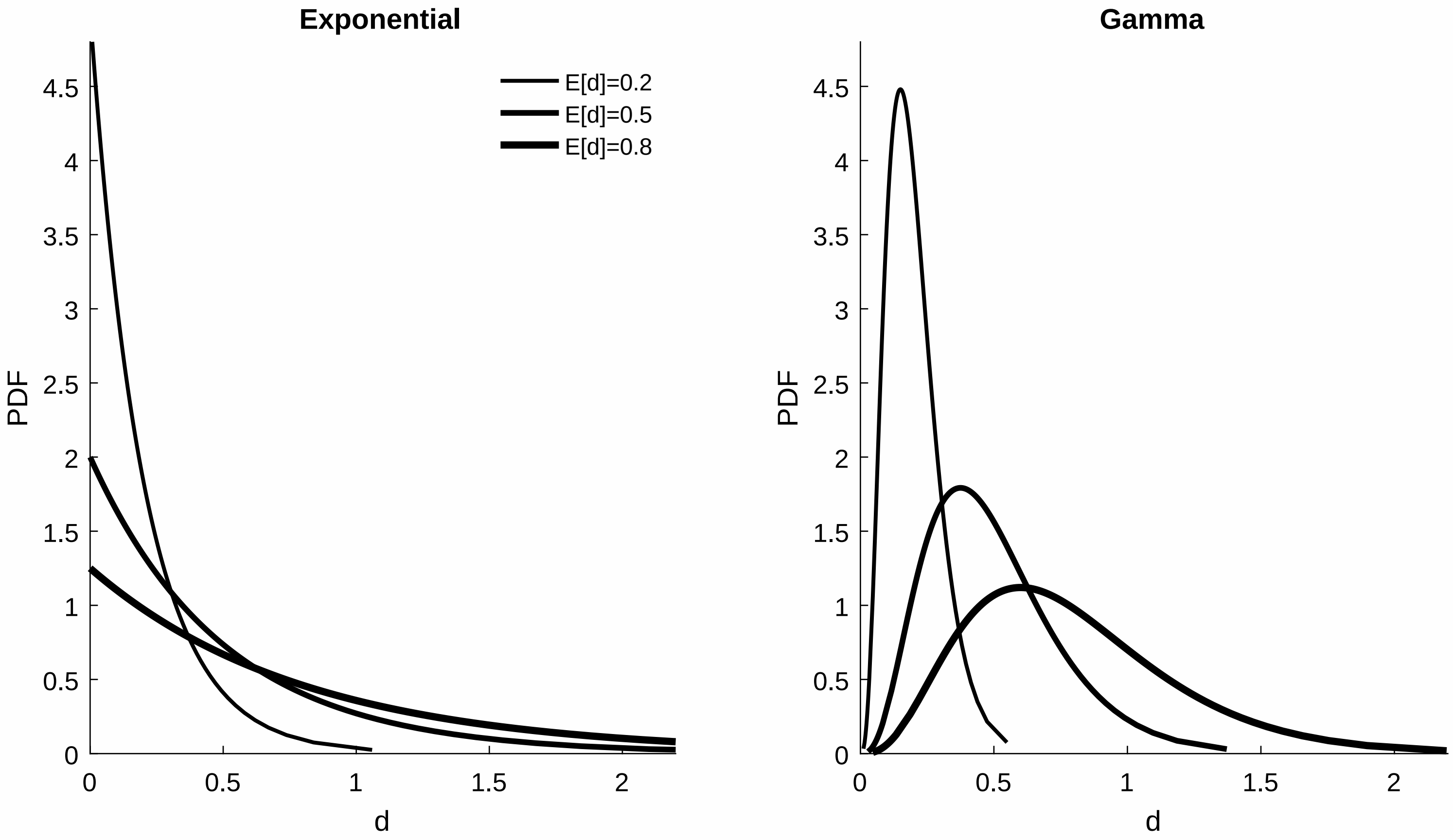

Fig B. Optimal α levels with varying effect sizes. Optimal α level, α_{optimal}, as a function of the base rate of true effects, π, the distribution of true effect sizes (fixed, exponential, or gamma), and the payoffs associated with false positives, $\mathcal{P}_{fp}$, and false negatives, $\mathcal{P}_{fn}$. For all distributions, the mean effect size was $d = 0.5$. There are similarly small differences between distributions in the analogous curves for $d = 0.2$ and $d = 0.8$.

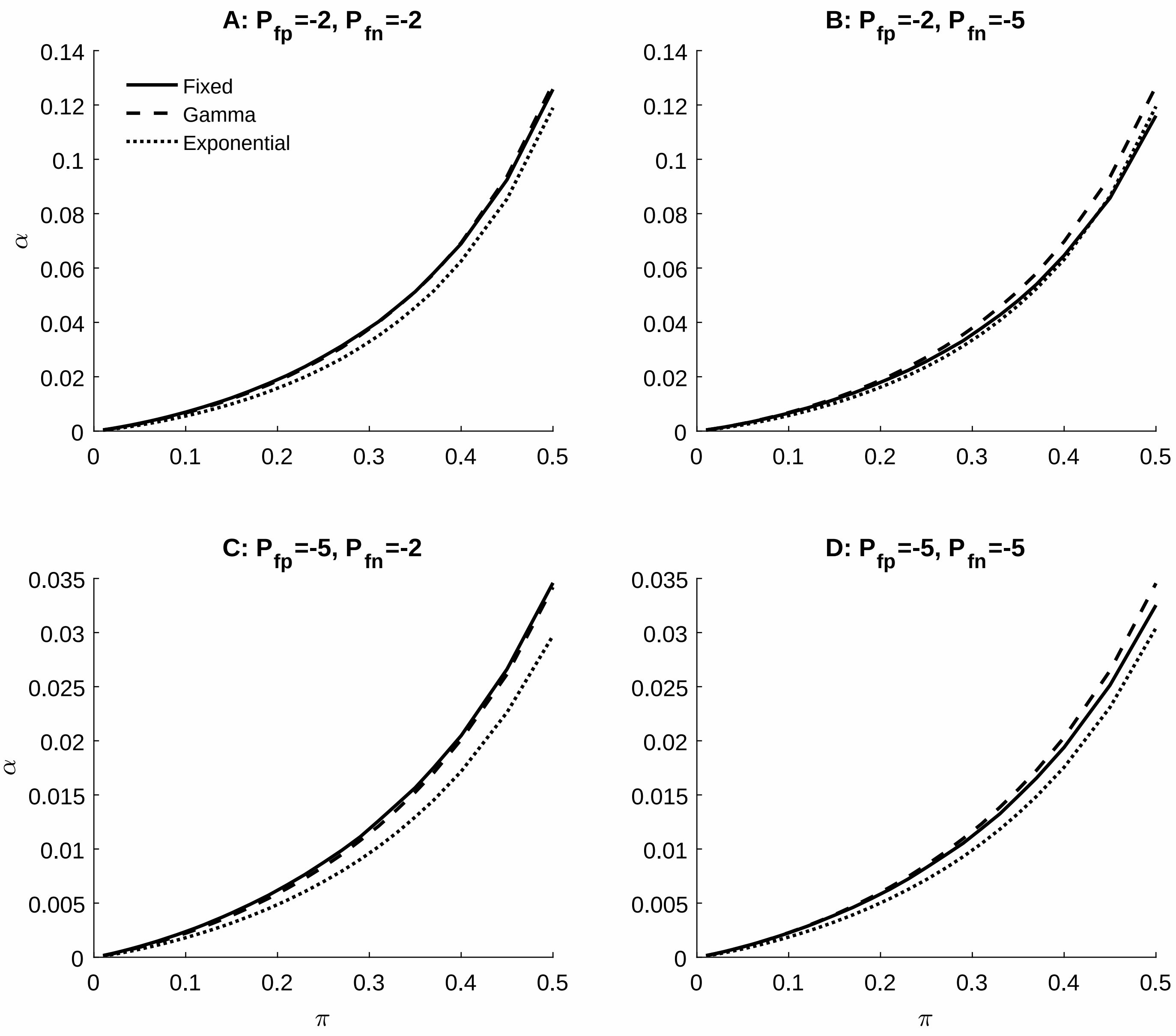

Fig C. Optimal sample sizes with varying effect sizes. Optimal sample size, $n_{\text{s,optimal}}$, as a function of the base rate of true effects, π, the distribution of true effect sizes (Fixed, Gamma, or Exponential), and the payoffs associated with false positives, $\mathcal{P}_{fp}$, and false negatives, $\mathcal{P}_{fn}$. For all distributions, the mean effect size was $d = 0.5$. There are similarly small differences between distributions in the analogous curves for $d = 0.2$ and $d = 0.8$.

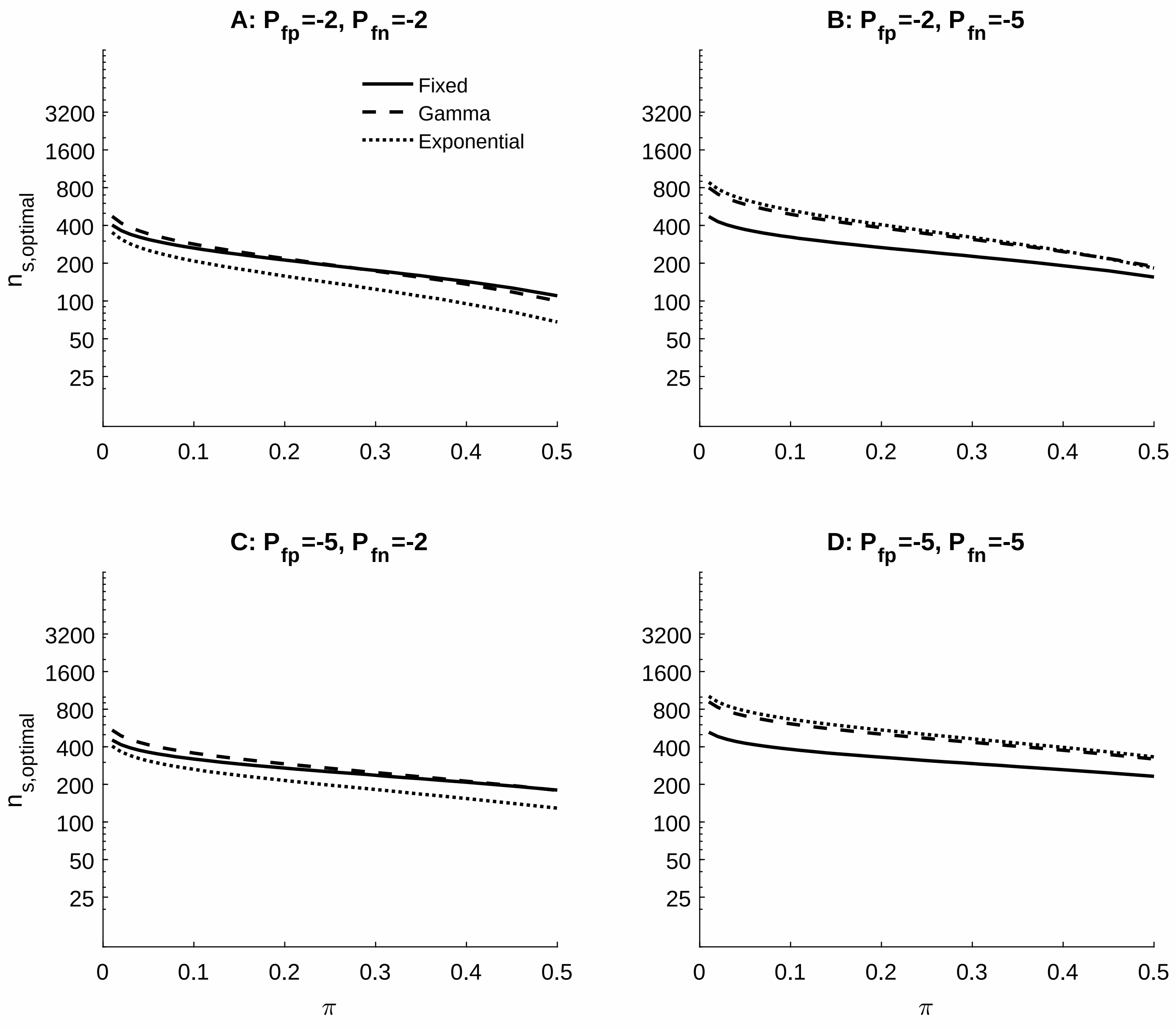

Supplement: S2 Appendix — (PDF) [file pone.0208631.s002.pdf]
